# A programmable CRISPR/Cas9-based phage defense system for *Escherichia coli* BL21(DE3)

**DOI:** 10.1186/s12934-020-01393-2

**Published:** 2020-07-03

**Authors:** Li Liu, Dongdong Zhao, Lijun Ye, Tao Zhan, Bin Xiong, Muzi Hu, Changhao Bi, Xueli Zhang

**Affiliations:** 1 University of Sciences and Technology of China, Hefei, 230026 P R China; 2 Tianjin Institute of Industrial Biotechnology, Chinese Academy of Sciences, Tianjin, 300308 P R China; 3 Key Laboratory of Systems Microbial Biotechnology, Chinese Academy of Sciences, Tianjin, 300308 P R China

**Keywords:** CRISPR/Cas9, *E. coli* BL21, Phage

## Abstract

*Escherichia coli* BL21 is arguably the most popular host for industrial production of proteins, and industrial fermentations are often plagued by phage infections. The CRISPR/Cas system is guided by a gRNA to cleave a specific DNA cassette, which can be developed into a highly efficient programable phage defense system. In this work, we constructed a CRISPR/Cas system targeting multiple positions on the genome of T7 phage and found that the system increased the BL21’s defense ability against phage infection. Furthermore, the targeted loci on phage genome played a critical role. For better control of expression of CRISPR/Cas9, various modes were tested, and the OD of the optimized strain BL21(pT7cas9, pT7-3gRNA, prfp) after 4 h of phage infection was significantly improved, reaching 2.0, which was similar to the control culture without phage infection. Although at later time points, the defensive ability of CRISPR/Cas9 systems were not as obvious as that at early time points. The viable cell count of the engineered strain in the presence of phage was only one order of magnitude lower than that of the strain with no infection, which further demonstrated the effectiveness of the CRISPR/Cas9 phage defense system. Finally, the engineered BL21 strain under phage attack expressed RFP protein at about 60% of the un-infected control, which was significantly higher than the parent BL21. In this work, we successfully constructed a programable CRISPR/Cas9 system to increase the ability of *E. coli* BL21’s to defend against phage infection, and created a resistant protein expression host. This work provides a simple and feasible strategy for protecting industrial *E. coli* strains against phage infection.

## Introduction

*Escherichia coli* is a major host for laboratory and industrial production of proteins, among which the BL21 series of strains are the most popular because they can expresses heterologous proteins at high levels [1]. However, BL21 can be easy infected by bacteriophages which can lead to enormous economic losses in industrial production [2], and there are no ideal solutions for this problem to date [2].

The CRISPR-Cas system, comprising clustered regularly interspaced short palindromic repeats (CRISPR) and associated proteins (Cas), is a molecular adaptive immune system of prokaryotes. The sequences between the arrays of CRISPR repeats are called spacers [3, 4]. When prokaryotes were infected by phages or other foreign nucleotides, the CRISPR-Cas system takes up the invading DNA and integrates it into CRISPR loci, which functions as a record or ‘memory’ [5]. Phage resistance specificity is determined by the similarity between the spacer and phage sequences [6]. In some lytic phages, the products of early-injected genes can degrade host DNA, inhibit the synthesis of host RNA and proteins, and protect the phage DNA, after which the rest of the viral DNA is injected into the cell [7]. The most spacers are acquired during DNA injection, in which early-injected genomic regions provide more effective adaptive immunity than late-injected genomic regions [8]. These loci within CRISPR can be transcribed and processed into small RNAs guiding Cas to cleave the phage chromosome [9].

Wild type *E. coli* MG1655 strains have a CRISPR-Cas3 system, which belongs to type I CRISPR-Cas, comprised of CRISPR, Cascades (CRISPR associated complex for antiviral defense proteins) and Cas3 [10]. The complete CRISPR-Cas3 system can target different sites on the genomes of *E. coli* phages such as λ, T7, or T4, and lead to a delay in the growth of phage progeny in the infected culture [11]. However, *E. coli* BL21 does not carry any CRISPR-Cas system according to its genomic sequence, and also according to predictions done using the CRISPRs web server (http://crispr.i2bc.paris-saclay.fr/?tdsourcetag=s_pcqq_aiomsg), suggesting that the sensitivity of BL21 to phages might due to its lack of adaptive immunity.

On the other hand, CRISPR/Cas9, a type-II CRISPR-Cas system, is the most widely applied type due to its relatively simple functional mechanism compared with other CRISPR systems. CRISPR/Cas9 only requires a guide RNA or crRNA/tracrRNA duplex with a Cas9 protein to function [3]. The protospacer adjacent motifs (PAMs) are utilized to make CRISPR–Cas9 distinguish between the self-target and non-self-target protospacers [12]. The CRISPR/Cas9 system was used to target human and plant viruses [13]. Some CRISPR/Cas9 based approaches were developed to defend against specific human viruses as a potential antiviral strategy for clinical applications [14]. For example, an SaCas9/gRNA system was programed to specifically target HIV-1 provirus and suppress HIV-1 infection in Jurkat C11 cells [15]. CRISPR/Cas9 was also employed to target plant viruses, which provided a more versatile viral resistance in important crops [16]. Class II CRISPR systems include Cas13a other than cas9 [17]. CRISPR/Cas13a (C2c2) was used to engineer interference against the RNA-based Turnip Mosaic Virus (TuMV) in plants [18]. FnCas9 and C2c2 were programed to break down the genome of geminiviruses in plants, providing resistance against virus infection by consistently reducing virus accumulation [19]. CRISPR/Cas may provide novel and reliable approaches to control geminiviruses and other ssDNA viruses such as Banana bunchy top virus (BBTV). Recently, PAC-MAN (Prophylactic Antiviral CRISPR in huMAN cells) was developed base on CRISPR-Cas13 system, which can effectively degrade RNA of SARS-CoV-2 sequences and live influenza A virus (IAV) in human lung epithelial cells [20]. PAC-MAN has the potential to become an important pan-coronavirus treatment by safe and effective respiratory tract delivery. Some phages with anti-CRISPR proteins can circumvent the CRISPR interference of their hosts by binding to and inactivating either the CRISPR complex or the executor nuclease [21]. However, anti-CRISPR systems in viruses are rare. Similarly, microbes employed in the fermentation industry often suffer from various bacteriophages, which makes a programmable CRISPR/Cas9-based phage defense system highly desirable to improve the consistency of the fermentation process and hence improve the process economics.

In this work, we chose the genomic loci within genes ecoding T7 phage encoding tail tubular protein gp12, capsid assembly protein and 3.8 protein as the targets. The tail tubular protein gp12 forms the end of the tail of T7 phage, including conical tube, nozzle, and small extensions below the fibers, which is important for viruses to inject their genomes into the bacterial cytoplasm [22]. The capsid assembly protein is a predicted protein for assembly of phage shell [23, 24]. And the 3.8 protein we selected is non-functional [24]. Our work designs a simple and programable CRISPR/Cas9 system to increase the ability of the widely used *E. coli* BL21 to defend itself against phage infection, producing a programmable resistant industrial host.

## Materials and methods

### Bacterial strains and culture conditions

All strains used in this work (Additional file 1: Table S1) are derived from *E. coli* BL21(DE3), and were cultured at 37 °C or 30 °C in Luria–Bertani medium (LB, 10 g/L, tryptone, 5 g/L yeast extract, 10 g/L NaCl) with apramycin (50 μg/ml) and chloramphenicol (30 μg/ml) or kanamycin (50 μg/ml) or ampicillin (50 μg/ml) when necessary.

### Plasmid construction

We used j5 DeviceEditor [25] to design DNA primers (Additional file 1: Table S3). DNA fragments were PCR-amplified using prime star (Takara, Japan) or Phusion polymerase (NEB, USA). BsaI restriction endonuclease and T4 ligase were purchased from Thermo-Fisher Scientific (USA). All the plasmids (Table 1) used in this study were constructed via Golden Gate method [25].

**Table 1:** The plasmids used in this study

Plasmids	Characteristics	
pCas9	Cas9 with constitutive promoter, Cm	Lab stock (Jiang et al. 2013)
pTFG025	Arabinose operon, Cas9, Km^r^	Lab stock
pT7-Cas9	Cas9 with T7 promoter, Amp	Lab stock
pgRNA	Plasmid for gRNA expression	Lab stock
pTgRNA	Derived from pgRNA targeting tail tubular protein B gene, Apr	This study
pCgRNA	Derived from pgRNA targeting capsid assembly protein gene, Apr	This study
p3.8gRNA	Derived from pgRNA targeting 3.8 protein gene, Apr	This study
p3gRNA	Derived from pgRNA targeting tail tubular protein B, capsid assembly protein and 3.8 protein, Apr	This study
pT7-TgRNA	Derived from pTgRNA, replacing the constitutive promoter by T7 promoter, Apr	This study
pT7-CgRNA	Derived from pCgRNA, replacing the constitutive promoter by T7 promoter, Apr	This study
pT7-3.8gRNA	Derived from p3.8gRNA, replacing the constitutive promoter by T7 promoter, Apr	This study
pT7-3gRNA	Derived from p3gRNA, replacing the constitutive promoter by T7 promoter, Apr	This study
pBBR1MCS2	broad-range host vector used for conjugation, Km^r^	lab stock
prfp	derived from pBBR1MCS2, BBa_J23100-*rfp*, Km^r^	Lab stock

We chose spacer sequences within the T7 phage tail tubular protein gp12, capsid assembly protein, and 3.8 protein genes as the N20 sequences of gRNA (Additional file 1: Table S2). The p3gRNA is assembled by three fragments from pTgRNA, pCgRNA and p3.8gRNA. The backbone containing TgRNA were amplified with primers 3gRNA-T-F and 3gRNA -T-R using pTgRNA as a template. The other two fragments are amplified by primers 3gRNA-C-F and 3gRNA-C-R and primers 3gRNA-H-F and 3gRNA-H-R, respectively. For the way of construction of pT7-3gRNA is same as the p3gRNA.

The strains carrying spacers targeting the phage DNA were named follows: strain name (the plasmid of cas9, the plasmid of gRNA, and prfp plasmid). For example, BL21(pT7cas9, pTgRNA, prfp) refers to BL21 carrying a spacer targeting the tail tubular protein gp12(T)gene of the phage.

### Assay for determining the efficiency of the CRISPR/Cas9-based phage defense system

The growth of bacterial cultures with or without phage infection was continuously monitored using an Infinite M200 PRO instrument (Tecan, Switzerland). Each experiment was repeated three times. Induced (0.05 mM IPTG) or uninduced cultures were grown in LB at 37 or 30 °C to an OD_600_ ≈ 0.8, which is easy to assay CRISPR/cas9 interference. BL21 contains pcas9 and pT7-cas9 should be cultured at 37 °C, while containing pBad-cas9 is cultured at 30 °C. Then, 10 mL cultures were infection with 1 or 10 μl of phages from a stock solution with a concentration of 10^8^/mL. Cell growth was continued for 4 h at 30 °C and 250 rpm.

To determine the number of living bacterial cells contain pT7-cas9, pT7-gRNA and prfp after phage infection, 100 μl aliquots of serial dilutions of infected or uninfected cultures were spread on LB plates containing 100 g/mL ampicillin, 50 mg/mL apramycin, and 50 mg/mL kanamycin and grown overnight. The aliquots were taken at 4 h and 16 h, and the number of living bacteria was calculated based on the number of colonies visible the next day. Each experiment was repeated three times.

### Measurement of RFP fluorescence

To measure the fluorescence intensity of RFP expressed by bacterial cultures with or without phage infection, 100 μl of each bacterial culture was added to a separate well of a 96-well clear-bottom plate. RFP fluorescence was measured using an Infinite M200 PRO plate reader (Tecan, Switzerland) using an excitation wavelength of 585 nm and an emission wavelength of 620 nm. Each experiment was repeated three times.

## Results

### Expression of CRISPR/Cas9 with gRNA targeting the T7 phage genome increased the phage defense ability of *E. coli* BL21(DE3)

**Fig. 1:**
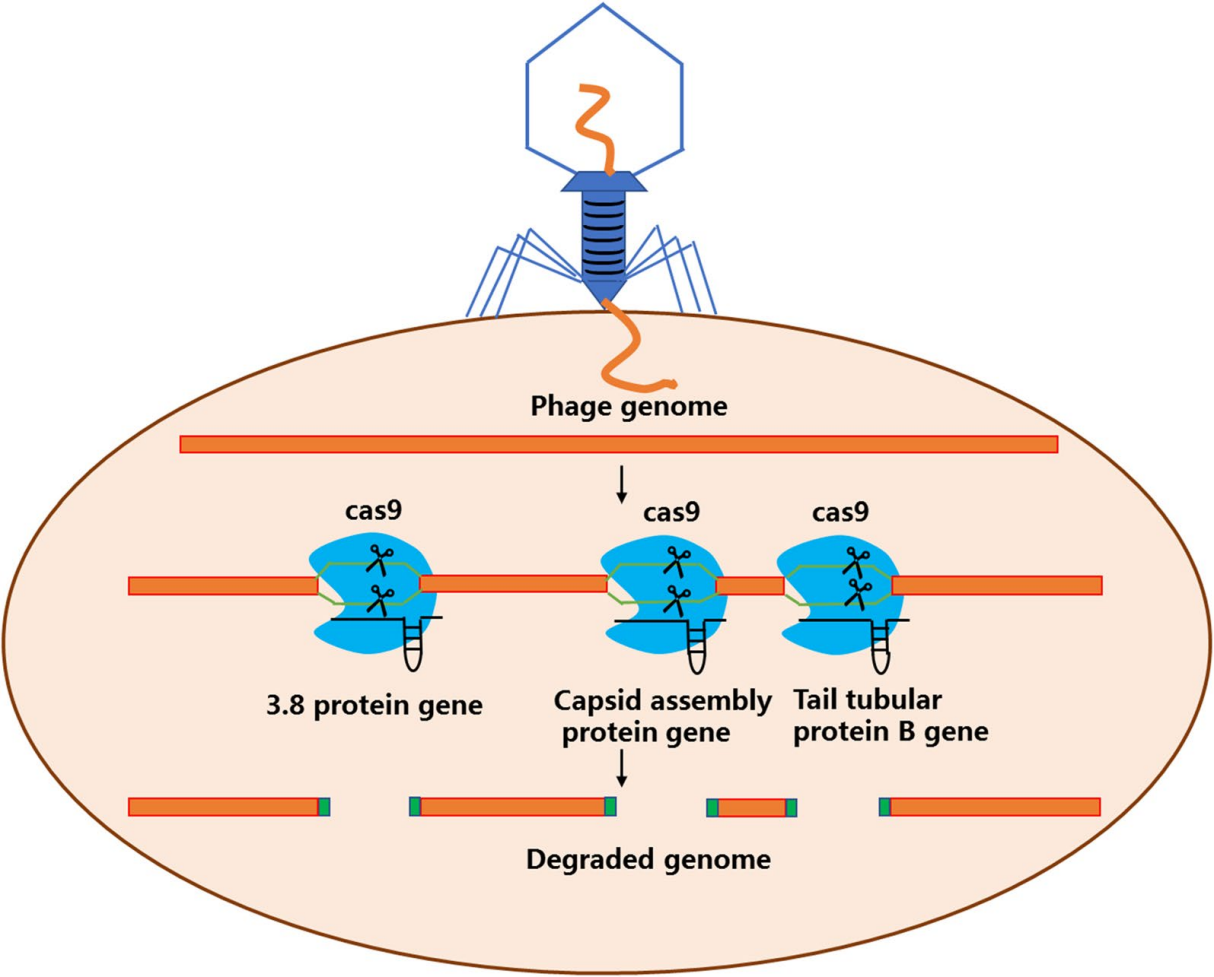
Schematic representation of BL21 carrying the CRISPR/Cas9 system programmed to cleave the genome of T7 phage

**Fig. 2:**
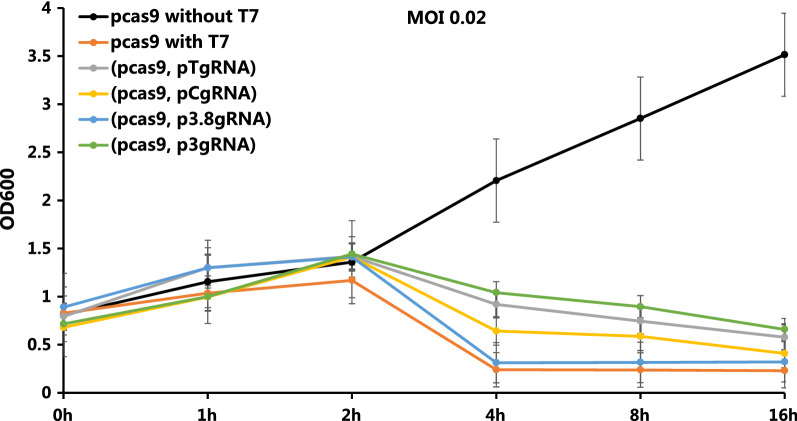
Growth curves of engineered BL21(DE3) strains with T7 phage infection. The data includes control cultures of the parental strain (without the system), with and without phage infection

To determine whether programed CRISPR/Cas9 can increase the ability of BL21 to defend itself against bacteriophages, we designed three different N20s, respectively targeting the genes of the tail tubular protein gp12, capsid assembly protein and 3.8 protein in the T7 phage genome (Fig. 1). Four strains, BL21(pcas9, pTgRNA), BL21(pcas9, pCgRNA), BL21(pcas9, p3.8gRNA), and BL21(pcas9, p3gRNA) were constructed, among which plasmid p3gRNA contained gRNA of all three loci. We prepared phage stock solution and 1 μl of it contains about 10^10^ phage. At MOI 0.02, the results showed that the OD_600_ of BL21(pcas9, pTgRNA) and BL21(pcas9, p3gRNA) was significantly higher than that of BL21(pCas9) with phage infection (Fig. 2). However, the OD_600_ of BL21(pcas9, pCgRNA) and BL21(pcas9, p3.8gRNA) was not distinctly higher than that of the control (Fig. 2). These results indicated that programed CRISPR/Cas9 targeting the phage genome increased the ability of BL21 to defend itself against phage infection, and the targeted loci played a critical role.

### Optimization of the programmable CRISPR/Cas9 phage defense system

**Fig. 3:**
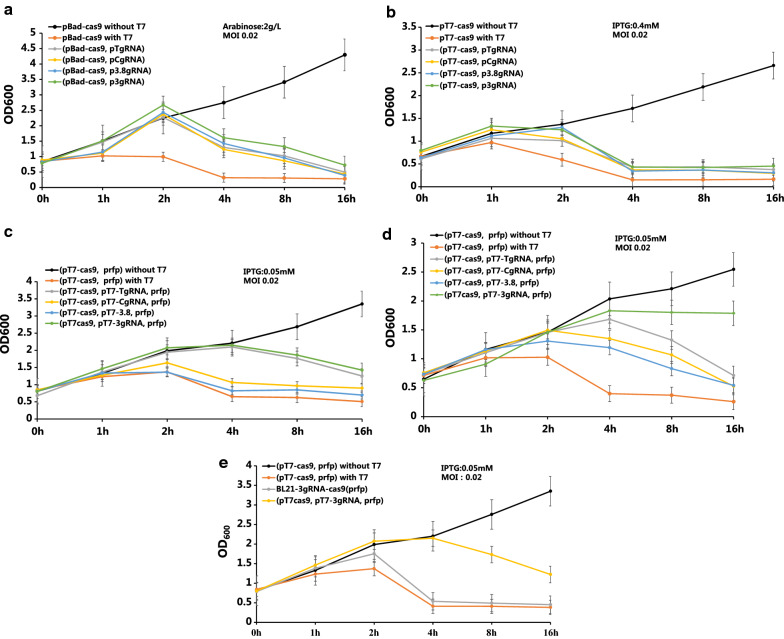
Growth curves of BL21(DE3) strains carrying the optimized CRISPR/Cas9 system with T7 phage infection. **a** Growth curves of strains based on the pBad promoter. Cultures were infected with 1 μl of phage stock in the presence 2 g/L arabinose. **b** Growth curves of strains based on the T7 promoter infected with 1 μl of phage stock with 0.4 mM IPTG. **c** Growth curves of strains based on the T7 promoter with 0.05 mM IPTG. **d** Growth curves of strains based on the T7 promoter infected with 10 μ of phage stock with 0.05 mM IPTG. **e** T7 based strains infected with 1 μl of phage stock with 0.05 mM IPTG

To better control the expression of CRISPR/Cas9, we replaced the constitutive promoter with the arabinose-inducible promoter (pBad promoter), resulting in strains BL21(pTFG025, pTgRNA), BL21(pTFG025, pCgRNA), BL21(pTFG025, p3.8gRNA) and BL21(pTFG025, p3gRNA). The OD_600_ of all the engineered strains after phage infection was higher than that of the control, and slightly higher than that of the strains with the constitutive promoter (Fig. 3a). The results illustrated that gene expression using the pBad promoter improved the performance of the CRISPR/Cas9 defense system, and suggested that inducible expression could be a better choice.

Considering that the BL21 series strains contain T7 polymerase, we replaced the pBad promoter with the T7 promoter to improve the expression of the CRISPR/Cas9 defense system. By changing the promoter of the Cas9 plasmid, strains BL21(pT7-cas9, pTgRNA), BL21(pT7-cas9, pCgRNA), BL21(pT7-cas9, p3.8gRNA) and BL21(pT7-cas9, p3gRNA) were constructed. However, when the concentration of the inducer IPTG was at 0.4 mM, the performance of the CRISPR/Cas9 defense system was not as good as that of the system expressed using pBad (Fig. 3b). To better modulate the expression of the CRISPR/Cas9 genes, the constitutive promoter for gRNA transcription was also replaced by the T7 promoter, and the IPTG concentration was reduced to 0.05 mM. The newly constructed strains had a very strong defense against phage infection, that the OD_600_ of BL21(pT7-cas9, pT7-TgRNA, prfp) and BL21(pT7cas9, pT7-3gRNA, prfp) after 4 h of phage infection was significantly improved to reach 2.0, which was similar to the control culture with no phage infection and obviously higher than the OD_600_ of BL21(pTFG025, p3gRNA) (Fig. 3c). To determine its performance with more severe phage infection, the phage concentration was increased 10-fold. Under such conditions, BL21(pT7cas9, pT7-3gRNA, prfp) still maintained an OD_600_ of around 1.7, slightly lower than that of the control (Fig. 3d) and the BL21(pT7cas9, pT7-3gRNA, prfp) in MOI 0.02, suggesting that the CRISPR/Cas9 defense system was robust in the presence of different concentrations of phage particles. After 4 h the defensive ability of CRISPR/Cas9 systems were not as obvious as that at early time points. At 16 h time points, the difference among different CRISPR/Cas9 systems were also reduced. However, the growth condition of our best engineered strain was still significantly better than the infected control strain.

We also integrated the pT7cas9 and pT7-3gRNA into the chromosome of *E. coli* BL21 at poxb locus by pCAGO [26] to construct the strain BL21-T7-3gRNA-T7-Cas9. However, the resistance of the integration strain against phage infection was not as good. The growth curve of BL21-T7-3gRNA-T7-Cas9 (prfp) was similar to the control with no CRISPR/Cas9, and significantly lower than that of the plasmid-based strain BL21(pT7cas9, pT7-3gRNA, prfp) (Fig. 3e). SDS-PAGE was used to analyze the expression of the Cas9 protein, which revealed that its content in BL21-3gRNA-cas9 was very low (Additional file 1: Fig. S1). The low expression may be due to only one chromosomal copy of the CRISPR/Cas9 gene being present in the integrated strain.

Furthermore, we performed a colony count to quantify the actual living cells in the samples, in addition to the OD_600_ readings. When BL21 was infected with the T7 phage, there were almost no surviving cells after either 4 h or 16 h of culture. By contrast, the control sample with no infection had a normal density of around 10^8^ to 10^9^ cells/mL. The strain BL21(pT7cas9, pT7-3gRNA, prfp) had a significantly higher survival rate, with 4.8 × 10^7^ and 2.7 × 10^7^ viable cells per mL after 4 h of infection with 1 μl and 10 μl of phage stock, respectively (Table 2, Additional file 1: Fig. S2). The viable cell numbers of both samples were only one magnitude lower than that of the strain with no infection, further proving the efficacy of the CRISPR/Cas9 phage defense system.

**Table 2:** 0.05 IPTG with 1 μ or 10 μ phage, proximate colony number

Strain	1 μ phage		10 μ phage	
	4 h	16 h	4 h	16 h
BL21(pT7cas9,prfp) without phage	6.65*10^8^	2.79*10^9^	7.7*10^8^	1.285*10^9^
BL21(pT7cas9, prfp) with phage	16	0	9	0
BL21(pT7cas9, p3gRNA, prfp)	4.8*10^7^	2.6*10^6^	2.7*10^7^	1.011*10^5^

### Increased protein expression of BL21 with the CRISPR/Cas9-based phage defense system

**Fig. 4:**
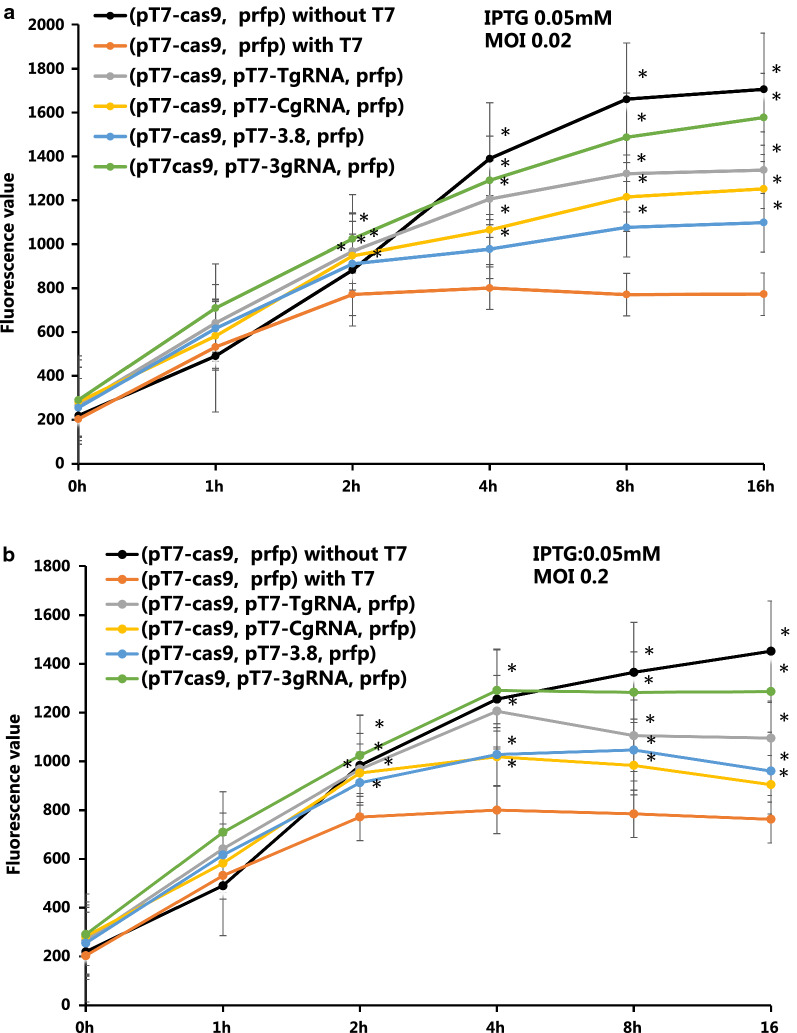
**a** Sensitivity of BL21 cells without the type II-A CRISPR-Cas system and of strains containing a type II-A CRISPR-Cas system to phage infection: BL21(pT7-cas9, pT7-gRNA, prfp) infected with 1 μ of phage stock, cultured at 30 °C in the presence of 0.05 mM IPTG. The protein levels of RFP correspond to the change of red fluorescence value; asterisks indicate significant differences compared with the BL21(pT7cas9, prfp) (*P < 0.05). **b** Sensitivity of BL21 cells without the type II-A CRISPR-Cas system and of strains containing a type II-A CRISPR-Cas system to phage infection: BL21(pT7-cas9, pT7-gRNA, prfp) infected with 1 μl of phage stock, cultured at 30 °C in the presence of 0.05 mM IPTG. The protein levels of RFP correspond to the changes of red fluorescence; asterisks indicate significant differences compared with the BL21 (pT7cas9, prfp) (*P < 0.05)

In order to evaluate the protein production capacity of BL21 stains with the CRISPR/Cas9 phage defense system, the plasmid prfp was transferred into the strains with CRISPR/Cas9 induced by T7 promoter. This plasmid was derived from the broad-host-range plasmid pBBR1MCS2, and the red fluorescent protein (*rfp*) gene driven by the constitutive promoter BBa_J23100 [27]. Rfp was expressed in the strains and the fluorescence value was measured after phage infection (Fig. 4a, b). For each strain, the fluorescence value of the vector control (pΔrfp) is about 0. It was found that all engineered strains performed better than the control strain with no CRISPR/Cas9. However, they were not as good as the control with no phage infection. The best performing strain was still BL21(pT7cas9, pT7-3gRNA, prfp) as in the OD_600_ experiments, which had a fluorescence value about 60% that of the un-infected control (Additional file 1: Fig. S3). We also calculated the value of FV/OD600. However, the value of all of the strains with the system was lower than the control strain, which indicated that the *rfp* gene might also be persistently translated when control strain was dead. The results support our hypothesis that the CRISPR/Cas9 defense system could be employed to protect BL21 and increase its protein production in bioprocesses with intractable phage infections in the equipment.

## Discussion

*Escherichia coli* especially *E. coli* BL21 is used to produce proteins which has important economic and social value [1]. Considering that phage outbreaks often brings significant financial losses of fermentation industries, we plan to construct a programmable CRISPR/Cas9-based phage defense system to help *E. coli* against phage infection. It was found that *E. coli* strains with its original CRISPR–Cas3 system could target various positions in the genome of bacteriophage T7 to resist the infection [28], and CRISPR immunity works like abortive infection mechanisms [29, 30]. For development of the defense system, at first, we tested the effect of different target loci. While the location of targeted loci played a critical role, it was not a surprise that simultaneous cleavage at multiple loci by CRISPR/Cas9 had the best defense effect. By manipulating with different promoters, induction patters and location of the DNA expression cassette, we found that plasmid based on expression with T7 for both cas9 and gRNA gave the best performance, basically indicated that phage defense with CRISPR/Cas9 required the strongest expression of the functional parts. At the MOI (multiplicity of infection) of 0.2, the OD of BL21(pT7-cas9, pT7-3gRNA, prfp) reach about 2.2 at 4 h which is higher than previous study with an culture OD of about 0.8 [28]. Furthermore, our result shows that the OD of BL21(pT7-cas9, pT7-3gRNA, prfp) can reach about 1.8 at 4 h at the MOI of 2.

Even with our strongest system, pT7-3gRNA, the infected phage was still not completely eliminated. The reason that CRISPR/Cas9 didn’t completely kill phage is probably due to the active recombination of phage genome during replication [30, 31], which resulted mutated genome to escape recognition of Cas9/gRNA. Finally, we obtained a functional phage defense system which significantly increased survived cell after phage infection, although the living cell count CFU was still 1 to 2 magnitudes lower than control strain with no phage infection, which indicated that the programmable CRISPR/Cas9-based phage defense system still has some space for improvement for industrial relative applications. Since our result revealed the importance of target location, one strategy might be scanning the whole phage genome for sensitive sites which gave the best cleavage efficiency by CRISPR/Cas9.

In this work, we successfully constructed a programable CRISPR/Cas9 system to increase the ability of *E. coli* BL21 to defend itself against T7 phage infection, thus creating a protein expression host with better performance. Since almost all phages have to inject their genomic DNA into the bacterial cells to complete their life cycle, this strategy could be applied for other phages. The only consideration is the forms of phage genomes, different CRISPR should be selected. The work provides a simple and feasible strategy for engineering industrial *E. coli* strains that are protected against phage infection.

## Supplementary information

**Additional file 1: Table S1** Strains used in this study. **Table S2** the properties of T7 phage. **Table S3** Oligonucleotides used in this study. **Figure S1** Agarose gels of cas9. **Figure S2** The number of living bacterium. **Figure S3** The shake flasks of cultures.

## Data Availability

We provide supporting and necessary data for publication of the article. All supporting data is present in the article and Additional file 1. The strains and plasmids associated with this work will be made physically available by the authors upon reasonable request.
