# Supplementary material for: A programmable CRISPR/Cas9-based phage defense system for Escherichia coli BL21(DE3)
Source: Microb Cell Fact. 2020 Jul 3;19:136. doi: 10.1186/s12934-020-01393-2 (PMC7332536; doi:10.1186/s12934-020-01393-2)
Supplement: Supplementary file 1 — Additional file 1: Table S1 Strains used in this study. Table S2 the properties of T7 phage. Table S3 Oligonucleotides used in this study. Figure S1 Agarose gels of cas9. Figure S2 The number of living bacterium. Figure S3 The shake flasks of cultures. [file 12934_2020_1393_MOESM1_ESM.doc]

**A programmable CRISPR/Cas9-based phage defense system**

**for *Escherichia coli* BL21(DE3)**

**Li Liu^a,b^, Dongdong Zhao^b,c^, Lijun Ye^b,c^, Tao Zhan ^b,c^, Bin Xiong^b,c^, Muzi Hu^b,c^, Changhao Bi^*b,c^, Xueli Zhang^*b,c^**

*^a^* *University of Sciences and Technology of China, Hefei 230026, P R China.*

*^b^ Tianjin Institute of Industrial Biotechnology, Chinese Academy of Sciences, Tianjin 300308, P R China.*

*^c^ Key Laboratory of Systems Microbial Biotechnology, Chinese Academy of Sciences, Tianjin 300308, P R China.*

^*^To whom correspondence should be addressed: X.Z. (email: zhang_xl@tib.cas.cn) or C.B. (email: bi_ch@tib.cas.cn)

**Table S1** Strains in this study

| Strains | Genotype and/or relevant features | Source or reference |
| --- | --- | --- |
| BL21(pBad-cas9, pTgRNA) | BL21 derivative containing a CRISPR-cas9 system with T spacer, modulate cas9 with Arabinose | This study |
| BL21(pBad-cas9, pCgRNA) | BL21 derivative containing a CRISPR-cas9 system with C spacer, modulate cas9 with Arabinose | This study |
| BL21(pBad-cas9, p3.8gRNA) | BL21 derivative containing a CRISPR-cas9 system with 3.8 spacer, modulate cas9 with Arabinose | This study |
| BL21(pBad-cas9, p3gRNA) | BL21 derivative containing a CRISPR-cas9 system with T，C and 3.8 spacer, modulate cas9 with Arabinose | This study |
| BL21(pcas9, pTgRNA) | BL21 derivative containing a CRISPR-cas9 system with T spacer | This study |
| BL21(pcas9, pCgRNA) | BL21 derivative containing a CRISPR-cas9 system with C spacer | This study |
| BL21(pcas9, p3.8gRNA) | BL21 derivative containing a CRISPR-cas9 system with 3.8 spacer | This study |
| BL21(pcas9, p3gRNA) | BL21 derivative containing a CRISPR-cas9 system with T，C and 3.8 spacer | This study |
| BL21(pT7-cas9, pTgRNA) | BL21 derivative containing a CRISPR-cas9 system with T spacer, modulate cas9 with T7 | This study |
| BL21(pT7-cas9, pCgRNA) | BL21 derivative containing a CRISPR-cas9 system with C spacer, modulate cas9 with T7 | This study |
| BL21(pT7-cas9, p3.8gRNA) | BL21 derivative containing a CRISPR-cas9 system with 3.8 spacer, modulate cas9 with T7 | This study |
| BL21(pT7-cas9, p3gRNA) | BL21 derivative containing a CRISPR-cas9 system with T，C and 3.8 spacer, modulate cas9 with T7 | This study |
| BL21(pT7-cas9, prfp) | BL21 derivative containing a CRISPR-cas9 system and prfp, modulate cas9 and gRNA with T7. | This study |
| BL21(pT7-cas9, pT7-TgRNA, prfp) | BL21 derivative containing a CRISPR-cas9 system with T spacer and prfp, modulate cas9 and gRNA with T7 | This study |
| BL21(pT7-cas9, pT7-CgRNA, prfp) | BL21 derivative containing a CRISPR-cas9 system with C spacer and prfp, modulate cas9 and gRNA with T7 | This study |
| BL21(pT7-cas9, pT7-3.8, prfp) | BL21 derivative containing a CRISPR-cas9 system with 3.8 spacer and prfp, modulate cas9 and gRNA with T7 | This study |
| BL21(pT7-cas9, pT7-3gRNA, prfp) | BL21 derivative containing a CRISPR-cas9 system with T, C and 3.8 spacer and prfp, modulate and cas9 and gRNA with T7 | This study |
| BL21-T7-3gRNA-T7-cas9 | pT7-cas9 and pT7-3gRNA are integrated into the *E. coli* BL21 at poxb locus | This study |
| E. coli DH5α | F^–^endA1 glnV44 thi-1 recA1 relA1 gyrA96  deoR nupG Φ80d*lac*ZΔM15 Δ(*lac*ZYAargF)U169,  hsdR17(r_K_^-^ m_K_+), λ | This study |

**Table S2** T7 Phage properities

| T7 phage | PAM | Position of protospacer in phage genome | Spacer sequence |
| --- | --- | --- | --- |
| Tail tubular protein B | AGG | 24857-24876 | CCAATCAATCAAGAACTTGA |
| Capsid assembly protein | AGG | 22304-22323 | GCAACTTGGTGAGCACGAAG |
| 3.8 protein | AGG | 11242-11261 | AACAATTCTATAAGGCTCCG |

**Table S3** Oligonucleotides used in this study

| **Primer** | **Sequence** | **Notes** |
| --- | --- | --- |
| gRNA-promoter-T-GAGT | CCAGGTCTCA GAGT CCAATCAATCAAGAACTTGAGTTTTAGAGCTAG | The primer pair used for cloning N20 of T into pgRNA vector |
| Promoter-gRNA-ACTC | CCAGGTCTCA ACTC AGATCTGACTCCAT  AACAGAGTACTCGC |
| gRNA-promoter-C-CGGT | CCAGGTCTCA CGGT GCAACTTGGTGAGCACGAAG GTTTTAGAGCTAG | The primer pair used for cloning N20 of C into pgRNA vector |
| Promoter-gRNA-ACCG- | CCAGGTCTCA ACCG AGATCTGACTCCATAACAGAGTACTCGC |
| gRNA-promoter-3.8p-GTGC | CCAGGTCTCA GTGC AACAATTCTATAAGGCTCCG GTTTTAGAGCTAG | The primer pair used for cloning N20 of 3.8 into pgRNA vector |
| Promoter-gRNA-GCAC | CCAGGTCTCA GCAC AGATCTGACTCCATAACAGAGTACTCGC |
| 3gRNA -T-F | CCAGGTCTCA GAGT CCAATCAATCAAGAACTTGAGTTTTAGAGCTAG | The primers used for constructing p3gRNA |
| 3gRNA -T-R | CCAGGTCTCAACCGAGATCTGACTCCATAACAGAGTACTCGC |
| 3gRNA -C-F | CCAGGTCTCACGGTGCAACTTGGTGAGCACGAAG GTTTTAGAGCTAG |
| 3gRNA -C-R | CCAGGTCTCAGCACAGATCTGACTCCATAACAGAGTACTCGC |
| 3gRNA -H-F | CCAGGTCTCAGTGCAACAATTCTATAAGGCTCCGGTTTTAGAGCTAG |
| 3gRNA -H-R | CCAGGTCTCAACTCAGATCTGACTCCATAACAGAGTACTCGC |
| T7-TgRNA-F | CCAGGTCTCACTATAGGGTAGCCCAATCAATCAAGAACTTGAGTTTTAGAGCT | The primer pair used for replacing constitutive promoter with T7 promoter in pTgRNA |
| T7-TgRNA-R | CCAGGTCTCAATAGTGAGTCGTATTAACAGGCTGACTTCAGGTGCTAGAGCA |
| T7-CgRNA-F | CCAGGTCTCATCACTATAGGGTAGCGCAACTTGGTGAGCACG | The primer pair used for replacing constitutive promoter with T7 promoter in pCgRNA |
| T7-CgRNA-R | CCAGGTCTCAGTGAGTCGTATTAACAGGCTGACTTCAGGTGC |
| T7-3.8gRNA-F | CCAGGTCTCAACTATAGGGTAGCAACAATTCTATAAGGCTCCGG | The primer pair used for replacing constitutive promoter with T7 promoter in p3.8gRNA |
| T7-3.8gRNA-R | CCAGGTCTCATAGTGAGTCGTATTAACAGGCTGACTTCAGGTGC |
| 3gRNA-PT7-T -F | CCAGGTCTCATGAGCGACAGATCGCTGAGATAGGTGC | The primers used for constructing pT7-3gRNA |
| 3gRNA-PT7-T -R | CCAGGTCTCATACAACGAACCACACTAGAGAACATACTGGC |
| 3gRNA-PT7-C -F | CCAGGTCTCATGTAATACGACTCACTATAGGGTAGCGC |
| 3gRNA-PT7-C -R | CCAGGTCTCACAACGAACCACACTAGAGAACATACTGGC |
| 3gRNA-PT7-H -F | CCAGGTCTCAGTTGTAATACGACTCACTATAGGGTAGCAAC |
| 3gRNA-PT7-H -R | CCAGGTCTCACTCAACGAACCACACTAGAGAACATACTGG |

kDa


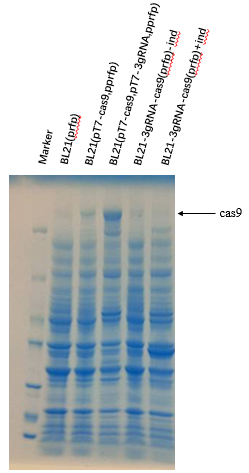


118

66

35

25

18

45

14

**Figure S1** the SDS PAGE showing the expression level of cas9 prepared from cells from BL21(prfp), BL21(pT7-cas9, prfp), BL21(pT7-cas9, pT7-3gRNA, prfp), BL21-3gRNA-cas9(prfp)-ind, BL21-3gRNA-cas9(prfp)+ind collected 4h post-infection are presented. The concentration of IPTG is 0.05mM.


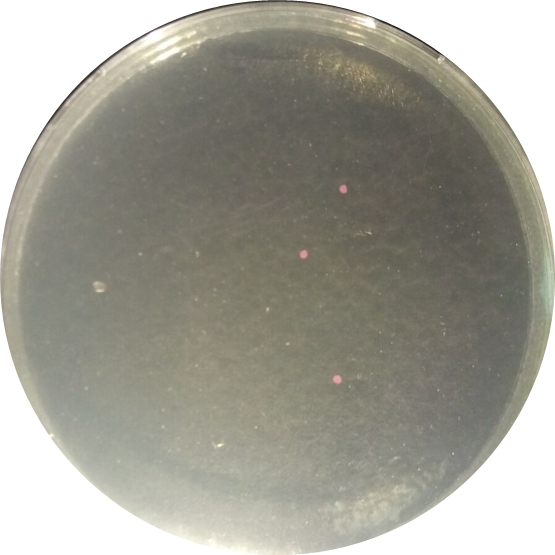

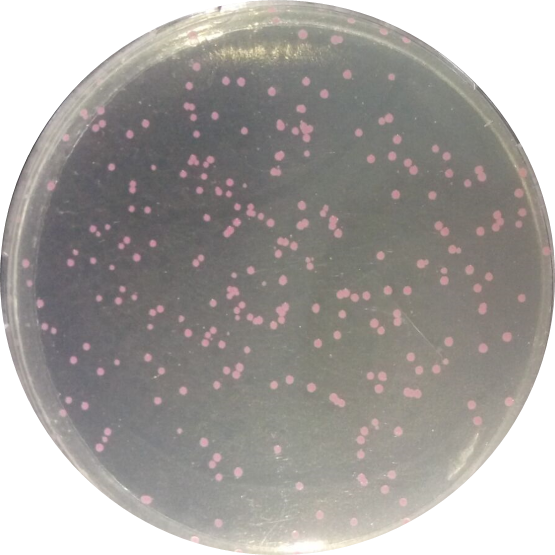

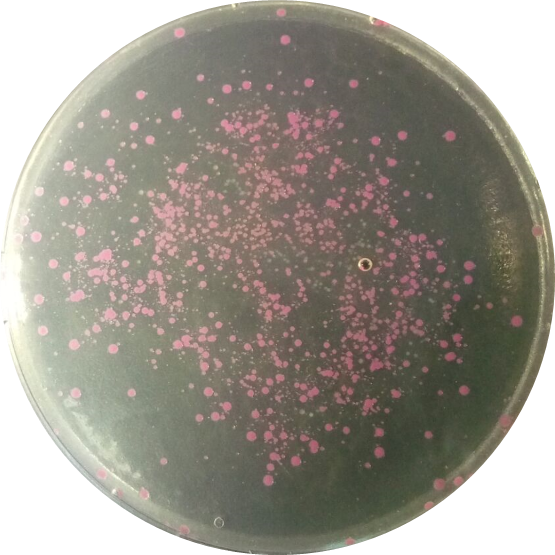

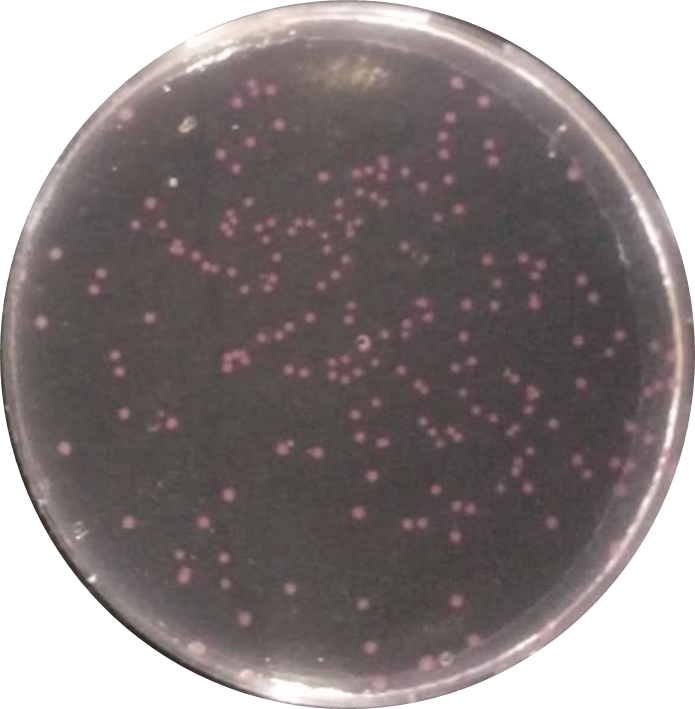


**A**

**B**

**C**

**D**

**Figure S2** Cultures was infected with 1 phage or not, in the presence 0.05mM IPTG. The aliquots were taken at 4h and 16h, and the number of living bacterium was determined. (A) The aliquots uninfected with phage were taken at 16h and diluted 10*^7^, the number of BL21(pT7cas9, prfp) is 2.58*10^9^. (B) The aliquots infected with phage were taken at 16h and not diluted, the number of BL21(pT7cas9, prfp) is 3. (C) The aliquots infected with phage were taken at 16h and dilute 10^3^, the number of BL21(pT7cas9, p3gRNA, prfp) is 6.82*10^5^. (D) The aliquots infected with 10μ phage were taken at 4h and dilute 10^5^, the number of BL21(pT7cas9, p3gRNA, prfp) is 2.08*10^7^.


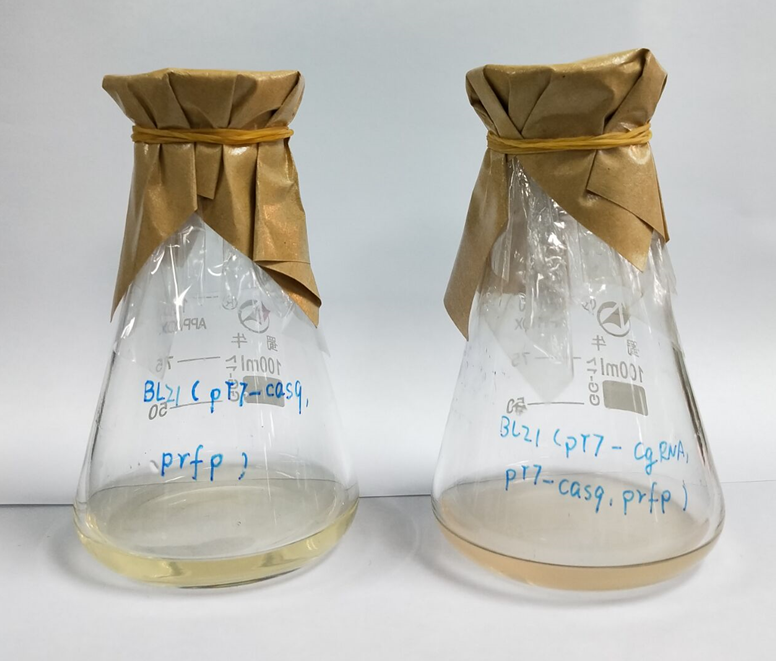

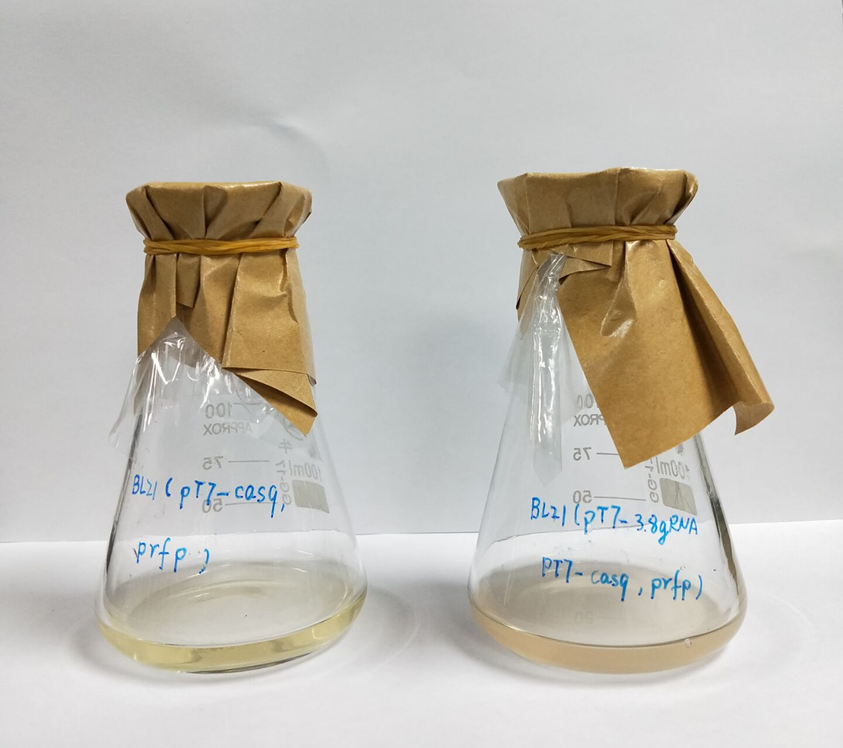


A

B

C

**
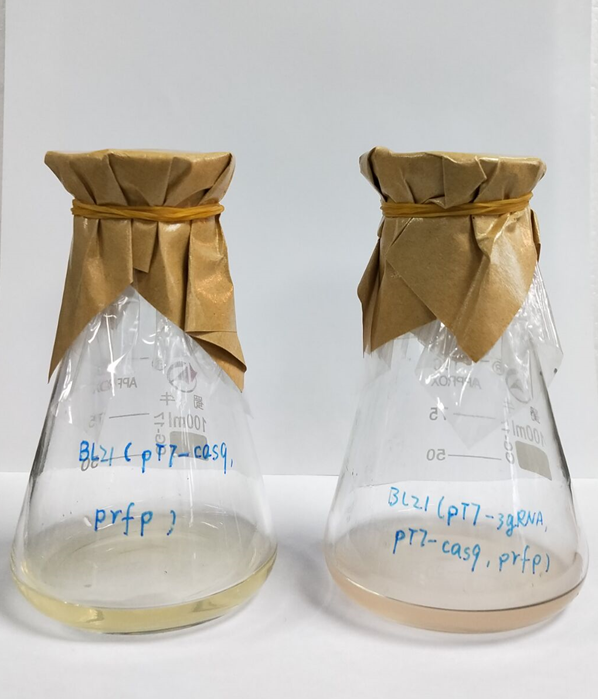
**

**Figure S3** Cultures was infected with 1u phage, in the presence 0.05mM IPTG. The shake flasks were taken at 4h.(A) Infection results of strain BL21(pT7cas9, prfp) and (p3.8gRNA, pT7cas9, prfp). (B) Infection results of strain BL21(pT7cas9, prfp) and (pCgRNA, pT7cas9, prfp). (C) Infection results of strain BL21(pT7cas9, prfp) and (p3gRNA, pT7cas9, prfp).
